# The forensiX Evidence Collection Tube and Its Impact on DNA Preservation and Recovery

**DOI:** 10.1155/2013/105797

**Published:** 2013-10-28

**Authors:** Alex M. Garvin, Ralf Holzinger, Florian Berner, Walter Krebs, Bernhard Hostettler, Elges Lardi, Christian Hertli, Roy Quartermaine, Christoph Stamm

**Affiliations:** ^1^Confarma France SARL, Zone Industrielle Canal d'Alsace, 68490 Hombourg, France; ^2^Institute of Chemistry and Biological Chemistry, Zurich University of Applied Sciences (ZHAW), Einsiedlerstrasse 31, 8820 Wädenswil, Switzerland; ^3^Prionics AG, Wagistrasse 27a, 8952 Schlieren, Switzerland

## Abstract

Biological samples are vulnerable to degradation from the time they are collected until they are analysed at the laboratory. Biological contaminants, such as bacteria, fungi, and enzymes, as well as environmental factors, such as sunlight, heat, and humidity, can increase the rate of DNA degradation. Currently, DNA samples are normally dried or frozen to limit their degradation prior to their arrival at the laboratory. In this study, the effect of the sample drying rate on DNA preservation was investigated, as well as a comparison between drying and freezing methods. The drying performances of two commercially available DNA collection tools (swab and drying tube) with different drying rates were evaluated. The swabs were used to collect human saliva, placed into the drying tubes, and stored in a controlled environment at 25°C and 60% relative humidity, or frozen at −20°C, for 2 weeks. Swabs that were stored in fast sample drying tubes yielded 95% recoverable DNA, whereas swabs stored in tubes with slower sample drying rates yielded only 12% recoverable DNA; saliva stored in a microtube at −20°C was used as a control. Thus, DNA sampling tools that offer rapid drying can significantly improve the preservation of DNA collected on a swab, increasing the quantity of DNA available for subsequent analysis.

## 1. Introduction

The generation of accurate DNA profiles is directly affected by the quantity and quality of the samples received at the laboratory. Therefore, it is important that the greatest quantity and best possible quality of sample arrives at the laboratory. Drying is a well-known method of DNA preservation; drying directly neutralises most of the important factors that cause DNA degradation, including bacteria, fungi, enzymes, and humidity [1–4].

Several different methods may be used to dry a sample, including active drying, passive drying, and air drying. Air drying is a form of passive drying in which the sample is not protected from outside contamination, and the likelihood of sample contamination and/or mix-up is increased. Passive drying is also dependent on climatic conditions; higher relative humidity in the external environment leads to a lower drying rate. Active drying is performed independent of the climatic conditions and in isolation from environmental influences. These drying methods also differ in the time required to fully dry a sample. In this study, the quantity of DNA which could be recovered from swabs was evaluated after a storage period of 14 days (taken as a representative period between forensic sample collection and analysis).

Human saliva was chosen as a source of DNA for this study, and as saliva naturally contains bacteria and fungi, it does not need to be contaminated artificially.

The study investigated the effect of the rate of sample drying on the quantity of recoverable DNA obtained from 2 commercially available sampling tools (swab and drying tubes). In addition, the influence of swab head material, the type of bacteria present, and the effect of drying on the viability of the bacteria were studied.

## 2. Materials and Methods

### 2.1. Forensic DNA Sampling Tools

The following two commercially available sampling tools were evaluated in this study:

1. the forensiX evidence collection tube, which contains an active desiccant system (SafeDry) and either a cotton swab or a nylon flocked swab (Prionics AG, Schlieren-Zurich, Switzerland);
2. the Sarstedt Forensic swab, which comes in a transport tube containing a ventilation membrane (passive drying) and employs a viscose swab (Sarstedt AG, Nümbrecht, Germany).

**Figure 1:**
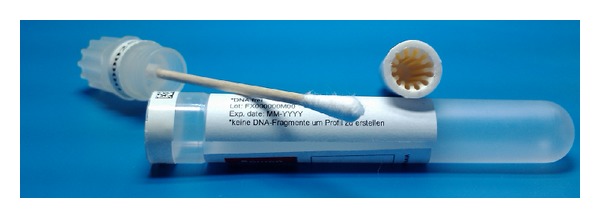
forensiX evidence collection tube, disassembled, with cotton swab and the SafeDry desiccant insert with a serrated inner surface.

**Figure 2:**
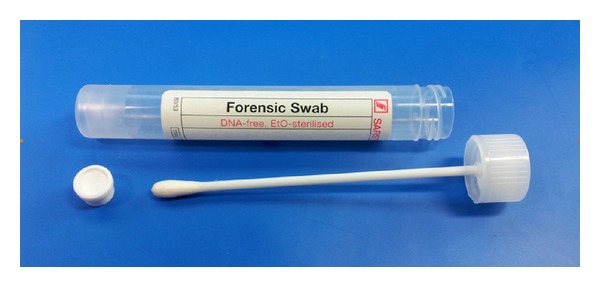
Sarstedt Forensic swab with disassembled ventilation membrane and viscose swab.

Each tool has a swab to collect the sample. The swab is placed in a tube and is dried by different drying mechanisms and at different rates. Photographs of the two tube types are shown in Figures 1 and 2.

The forensiX evidence collection tube uses a proprietary desiccant insert containing a molecular sieve drying agent in a polymer matrix (termed the “SafeDry” desiccant). This system actively dries the sample swab by absorbing all the water from the air space surrounding the sample. The rate of moisture uptake by the desiccant has been optimised through a serrated surface that creates a large inner surface area, and the desiccant is positioned around and near (ca. 2 mm) the swab head such that the swab head is surrounded by the desiccant. The forensiX tube isolates the sample from external climatic conditions with an airtight cap, protecting the sample from contamination.

The Sarstedt Forensic swab has a water vapour-permeable membrane attached to the base of the transport tube (ventilation membrane). This membrane allows the passive drying of the swab as water vapour diffuses from the swab to the end of the transport tube and through the membrane. As long as the external relative humidity is lower than that in the transport tube, the tube will lose water vapour. The effectiveness of this system is therefore highly dependent on the relative humidity of the environment external to the transport tube. Although it is not isolated from the external climatic conditions, the Sarstedt tube protects the sample from contamination via the membrane and a screw cap.

### 2.2. DNA Sample Material

Human saliva was selected as the sample type for the recovery of nuclear DNA. Saliva is a common nuclear DNA-yielding tissue found at crime scenes. Other relevant DNA-containing samples found at crime scenes include skin flakes, fingerprints, dandruff, blood, and semen.

### 2.3. Measurement of Swab Drying Rate in Collection Tubes

To measure the effectiveness of swab drying in the different collection tubes, 100 *μ*L (0.1 g) of distilled water was added to each swab type. Cotton swabs or nylon flocked swabs were used for the forensiX evidence collection tubes; only a viscose swab was available for the Sarstedt Forensic Swab. The wet swabs were placed in their respective tubes, sealed, and stored at 24 ± 2°C and at a relative humidity of 50 ± 5% for 14 days. The experiments were conducted using *n* = 5 samples for each type of collection tube.

At 30 minute intervals during the first 7 hours (420 minutes) of drying, the swabs were removed from their respective tubes and weighed to determine the quantity of water remaining on the swab. Thereafter, the swabs were weighed once a day using a Sartorius BP121S scale for a total of 14 days.

### 2.4. Saliva Preparation and Storage

For this series of experiments, 5 mL of saliva was collected from a single individual and was vortexed for 1 minute. As reported by Aps et al. [5] the number of leukocytes and epithelial cells present in human saliva can be determined using flow cytometry. The number of nucleated human cells (leukocytes and epithelial cells) in the saliva sample was determined to be 1,321 cells/*μ*L using a haemocytometer. Then, 100 *μ*L of this saliva stock, containing 132,100 nucleated human cells, was added to each swab using a Gilson P200 Pipetman. The estimated human nuclear DNA content of 100 *μ*L is 872 ng, as each diploid human cell contains 6.6 pg of nuclear DNA as reported by Lo et al. [6]. The stock was vortexed before each pipetting step. All samples were analysed in triplicate. Three positive controls consisting of 100 *μ*L of saliva placed directly in 1.5 mL microfuge tubes were also prepared. In total, 30 samples of 100 *μ*L of saliva each were prepared, using 3 mL of the 5 mL saliva stock.

Three positive controls consisting of saliva were placed directly in 1.5 mL microfuge tubes. Three Sarstedt swabs (in tubes) and 3 forensiX swabs (in tubes) also containing saliva samples were frozen at minus 20°C for 14 days.

The remaining sample swabs and tubes containing saliva (21 tubes in total) were placed in a temperature and humidity controlled room at Confarma France SARL and maintained at 25°C and at 60% relative humidity. This room was certified under ISO 9001 and cGMP for this purpose. The 60% humidity in this room was too low to permit water deposition on any objects placed in the room, and no visible water was present on any surfaces during the time of the experiment. The tubes containing the swabs were placed in the room for 14 days and were not disturbed during the entire time.

Comparisons were made between forensiX tubes with and without desiccant and Sarstedt tubes with and without passive drying. The desiccant was removed from 3 forensiX tubes, and the membranes of 3 Sarstedt tubes were covered with Scotch tape to block moisture diffusion out of the tubes. A total of 9 forensiX swabs and 9 Sarstedt swabs were prepared for this experiment: 3 tubes without desiccant/passive drying, 3 tubes with desiccant/passive drying, and 3 tubes as controls (frozen, −20°C).

Table 1 summarizes the range of prepared swabs and tubes and their storage conditions.

### 2.5. Saliva DNA Purification

Saliva nuclear DNA was prepared from the collected samples using the Qiagen QIAmp DNA Mini Kit (Art. No. 51304, Qiagen GmbH, Hilden, Germany). The buccal swab protocol was used for the swab samples, and the protocol for DNA purification from blood and bodily fluids was used for the three saliva controls (no swab or tube, samples 1–3). The DNA was eluted from the columns with 100 *μ*L of ATE buffer (10 mM Tris-HCl, 0.1 mM EDTA, 0.04% Sodium Azide; pH 8.3) a low-EDTA elution buffer optimised for long-term DNA storage.

### 2.6. Saliva DNA Quantification

Saliva DNA was quantified using the Quantifiler DUO Human DNA Quantification Kit from Life Technologies (Art. No. 4387746, Life Technologies Inc., Carlsbad, CA, USA). A standard curve was generated using 25 *μ*L reactions with human male DNA at 100,000 pg, 10,000 pg, 1,000 pg, 100 pg, and 0 pg per well. As specified by the DUO protocol, 2 *μ*L of sample DNA was added to 23 *μ*L of PCR mix/primers. Data were generated and analysed on an Applied Biosystems 7500 Fast Real Time PCR System.

### 2.7. Influence of Swab Material

To confirm that any differences in DNA recovery observed were due to the higher drying rate and not differences in the swab materials, an experiment was performed at Eurofins Medigenomix GmbH, Ebersberg, Germany, to compare the influence of the different swab head materials on DNA release.

A saliva stock was homogenised. 100 *μ*L of saliva was added to three cotton swabs (forensiX) and three viscose swabs (Sarstedt), and the swabs were dried in a cabinet dryer (not in a tube) at 56°C for 2 hours. The DNA on the swabs was extracted using the Qiagen QIAsymphony DNA Investigator Kit (Art. No. 931436, Qiagen N.V., Venlo, The Netherlands). Each extract was quantified twice using the Qiagen Investigator Quantiplex Kit (Art. No. 387016, Qiagen N.V., Venlo, The Netherlands).

### 2.8. Microbial Growth

Both the forensiX and the Sarstedt swabs were tested for viable microorganisms after 14 days of storage to investigate whether drying the saliva samples preserved the DNA by killing the associated microorganisms or by suspending their metabolism.

A 100 *μ*L sample of prepared human saliva (described in Section 2.4 above) was added to each swab which were then stored for 14 days under the conditions shown in Table 2.

All experiments were conducted using 3 swabs per assay. Cells were eluted from the swabs in 1 mL of distilled water each and then diluted 1 to 10 and 1 to 100 in distilled water. Then, 100 *μ*L of each dilution was spread on Sabouraud dextrose agar (SDA) plates and incubated at 22.5°C for 48 hours before performing colony counts.

### 2.9. Identification of Microbes Present on Human Saliva-Spiked Swabs

Following the study of microbial growth on human saliva spiked swabs, bacterial colonies were identified by mass spectrometry. Only colonies that gave a score above 2.0 (secure genus identification) using Biotyper 2.0 [7] were included in this study.

### 2.10. Effect of Sample Drying on Microbial Activity

To further investigate the influence of the drying process on bacterial growth, model experiments were conducted with the bacterium *Pseudomonas putida*. Although this bacterium is not found in saliva, it was chosen for this initial investigation because it is a stress-resistant species of bacteria with high rates of metabolism and growth [8].

Proliferating bacterial cultures have a higher adenosine triphosphate (ATP) content than that of metabolically inactive or dying bacteria [8]. Therefore, swabs were inoculated with a suspension of *Pseudomonas putida*, placed in forensiX evidence collection tubes with or without the fast drying desiccant, and the time-dependent growth of the bacteria was measured by determining the ATP level, which served as a marker for an increase or decrease in the metabolic activity of the total biomass.

*Pseudomonas putida* strain DSMZ 291 was incubated in a nutrient broth (BD Difco Nutrient Broth 234000, 8 g/L) at 27°C with mixing by rotation at 150 rpm. Incubation was stopped after 16 hours, and the cells were shown to be in the exponential growth phase, with an optical density of 1.5 at 600 nm and a total cell number of approximately 2 × 10^10^ per mL.

Cotton swabs were spiked with 100 *μ*L of this suspension and incubated at 27°C over a 24 hour period in the forensiX tubes both with and without desiccant. All experiments were conducted in triplicate.

The bacterial ATP content [9] was measured using the BioThema ATP Biomass Kit HS (product number 286-311, BioThema, Handen, Sweden). Following incubation, the swab tips were cut and placed in Eppendorf tubes filled with 1 mL of maximum recovery diluent (peptone saline diluent: 1 g peptone, 8.5 g NaCl, 0.3 g KH_2_PO_4_, 0.6 g Na_2_HPO_4_ (2H_2_O), and pH 7.0) and vortexed for 5 seconds. After 1 minute, each tube was vortexed again for 5 seconds and centrifuged (4 min at 16,000 ×g). Then, 0.5 mL of the supernatant was removed to measure the extracellular ATP content. To facilitate the extraction of ATP, 0.5 mL Extractant B/S (BioThema, Sweden) was added to each tube and vortexed for 5 seconds. After incubation for 10 minutes at room temperature, each tube was vortexed again, and then 0.2 mL of supernatant was removed to measure the total ATP content. The intracellular ATP content was calculated as the difference between the total and the extracellular ATP content. The ATP levels were measured and calculated according to the instructions provided with the BioThema kit.

## 3. Results

Two different forensic sampling tools were analysed for their capacity to effectively dry biological samples containing nuclear DNA. The most important parameter, the drying rate, was then correlated with the degree of DNA preservation.

### 3.1. Swab Drying Rates of Different Forensic Sample Collection Tubes

Two types of swab material (cotton and nylon) commercially available for the forensiX collection tubes were tested. A viscose swab was used with the Sarstedt tube.

**Figure 3:**
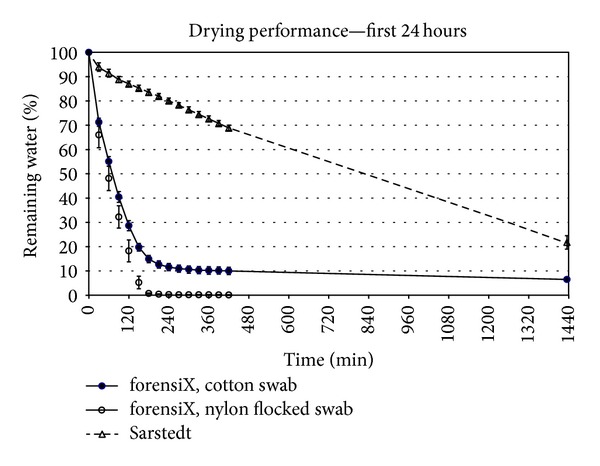
Drying curve for the initial 24 hours: the percentage of remaining water on swabs in collection tubes (*n* = 5) at 24 ± 2°C with a relative humidity of 50 ± 5%.

**Figure 4:**
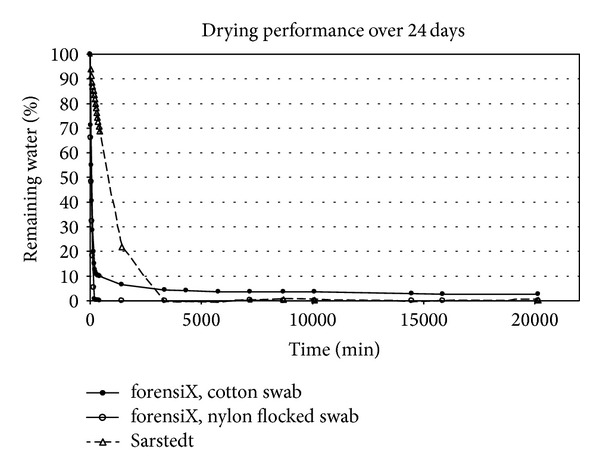
Drying curve over 14 days: the percentage of remaining water on swabs in collection tubes (*n* = 5) at 24 ± 2°C with a relative humidity of 50 ± 5%.

Figure 3 shows the percentage of water remaining on the swabs over the first 24 hours of incubation, and Figure 4 shows the percentage of water remaining over a total of 14 days, for each of the 3 types of tubes and swabs studied. The error bars in Figure 3 show the standard deviation.

Figures 3 and 4 show that the forensiX evidence collection tube provided the most rapid drying within the first 24 hours. During the first 2 hours, the drying rate achieved with the forensiX tubes was 0.68%/min for nylon flocked swabs and 0.60%/min for cotton swabs. The Sarstedt tube exhibited a drying rate of 0.083%/min, a rate at least 7 times slower than the forensiX tube.

In the forensiX tube, the nylon flocked swab (which was attached to a plastic stem) was completely dry within 4 hours, and the cotton swab (which was attached to a wood stem), dried to a level of 10% remaining water within 6 hours.

Under the experimental conditions of 24 ± 2°C and a relative humidity of 50 ± 5%, the swab in the Sarstedt tube dried completely in approximately 29 hours (extrapolated from the Sarstedt data in Figure 3).

### 3.2. Recovery Yields of DNA from Saliva after Storage in Different Sample Collection Tubes

The frozen saliva (control) samples yielded an average of 455 ng of DNA, or 52% of the estimated 872 ng of DNA in the 132,100 nucleated human cells present in the 100 *μ*L of saliva that was used as the starting material. This yield is similar to that found by Brownlow et al [10]. who used the Qiagen DNA Investigator Kit to isolate DNA from saliva-spiked cotton swabs.

Table 3 presents the results of the quantification assays performed on the DNA isolated from the 3 frozen saliva controls (samples 1–3) and from the swabs stored in tubes (samples 4–21). The yields obtained for the saliva stored on swabs are expressed as a percentage of the yield obtained from the 3 saliva control samples (stored in microfuge tubes).

The frozen forensiX and Sarstedt swabs yielded 72% and 54%, respectively, of the amount of DNA obtained from the frozen saliva control samples. 

Swabs stored in the forensiX tubes without desiccant (samples 10–12), and therefore with high moisture content remaining on the swab, yielded only 4.8% of the DNA obtained from the saliva controls, whereas identical samples with desiccant (samples 13–15), and therefore with low moisture content on the swab, provided a 95% recovery yield compared with the saliva controls.

Swabs stored in Sarstedt tubes with the sealed ventilation membranes (samples 16–18), in which a higher moisture content remained on the swab, had low recovery yields, averaging ca. 1% of the positive controls. This demonstrates that when the membrane is blocked and little moisture can leave the tube, DNA degradation is maximised. When the Sarstedt ventilation membrane was not covered with tape, the swabs (samples 19–21) gave recovery yields averaging 12% of the saliva controls. Therefore, the amount of DNA obtained with the tubes using a high performance desiccant was 7.9 times (431 ng/56 ng) higher than the amount obtained with storage tubes using a ventilation membrane.

### 3.3. The Influence of Swab Material on the Quantity of Recoverable DNA

The average DNA yields shown in Table 4 are based on *n* = 6 DNA quantifications.

The results summarised in Table 4 show that the retention of DNA is not significantly affected by the type of swab material used.

### 3.4. The Effect of Drying on Microbial Growth in Human Saliva-Spiked Swabs

Table 5 shows the number of colonies present on the SDA plates for the dilutions of cells eluted from the 3 types of swabs.

No viable microorganisms were detected on the forensiX swabs, whereas the Sarstedt swabs contained a considerable number of colony-forming microbes.

### 3.5. Identification of Microbes Present on the Sarstedt Tubes

Overall, 32 of the 40 colonies that were tested had a score above 2.0. The identification of these bacteria at the genus level is presented in Table 6. Two colonies were fungal, as determined by microscopic analysis, and were not analysed further.

All of these genera are members of the Enterobacteriaceae family of gram-negative bacteria and have been found in human fluids and tissue [11].

### 3.6. Effect of Sample Drying on Microbial Activity

**Figure 5:**
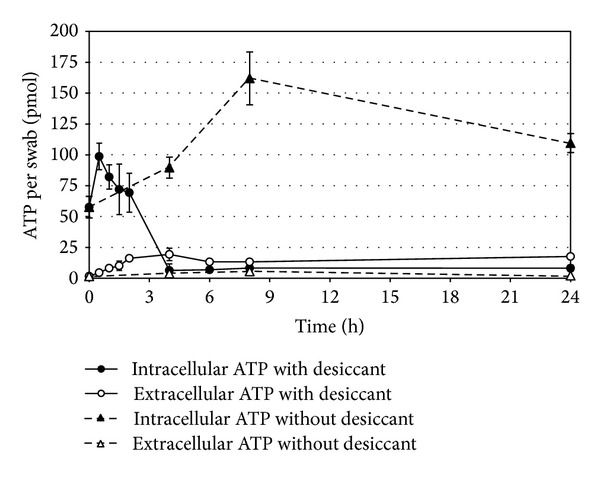
Temporal changes in the ATP content of *P. putida* during 24 hours on swabs in forensiX tubes with or without a desiccant (*n* = 3).

Temporal changes in the intracellular and extracellular ATP content of the swabs in a forensiX drying tube with and without desiccant are shown in Figure 5.

In the tubes without desiccant, the intracellular ATP content increased during the first 9 hours and remained relatively high for the remaining 24 hour incubation period. In contrast, the tubes with desiccant showed only a short-lived increase in intracellular ATP levels during the first 30 minutes before decreasing to low levels within approximately 4 hours. Extracellular ATP levels remained low throughout the 24 hour incubation period for both tubes.

The extracellular ATP levels were slightly higher in the desiccant tubes than in the desiccant-free tubes. This has been interpreted to represent the bacterial release of ATP during the drying process (i.e., cell disintegration).

## 4. Discussion

Biological evidence, whether obtained at a crime scene, from a rape victim or from a suspect, is often collected using a swab, and this collection is usually the first step in a long process of obtaining the DNA profiles of possible perpetrators. Once the DNA-containing sample is on the swab, it must then be stored in a way to prevent the degradation and contamination of the sample before the DNA can be extracted and analysed. Much of the degradation occurs due to the presence of bacteria and fungi that will inevitably be collected with the sample. During sample storage—which can be days, weeks, or even years—biological degradation can occur. This degradation is most problematic for samples containing only small quantities of cellular material, for example, touch DNA samples. Storage of a swab in a tube protects a sample from the risk of external contamination. Different preservation mechanisms can be used to halt the growth of bacteria present in a sample. For example, samples can be dried by either active or passive drying, or samples can be cooled or frozen.

This study investigated the effects of two drying systems on the DNA yield from saliva samples stored for 14 days at room temperature (25°C) and at 60% relative humidity. The key factor for preserving DNA was determined to be not the final extent of drying but rather the rate of drying of the sample. The faster a sample is dried, the greater the amount of recoverable DNA obtained.

The drying rate of two commercially available swabs in their respective tubes was measured. The results showed that the remaining water on a swab was reduced to 20% within 2.5 hours using the forensiX evidence collection tube and within 25 hours with the Sarstedt Forensic swab. The wood stem used with one of the forensiX swabs is the likely reason for the higher remaining water level on the swab when compared with the forensiX nylon-flocked swab. The wood stem absorbed water from the swab when it was moistened, increasing the time required to fully dry the swab and wood stem. Importantly, further experiments showed that the remaining 10% water had no significant effect on the recovery of DNA from the sample.

Saliva DNA yields of 95% were obtained with the forensiX evidence collection tube and 12% using the Sarstedt Forensic swab. Thus, the swabs spiked with 100 *μ*L of saliva suffered almost no DNA degradation when stored in the forensiX tube, whereas swabs stored in Sarstedt tubes suffered significant degradation. Frozen forensiX and Sarstedt swab systems yielded 72% and 54% DNA, respectively, showing that fast drying is superior to freezing.

This study investigated the effect of drying on the quantity of DNA that could be recovered from swabs. As the quantity of nuclear DNA used in these experiments was large, a confirmatory analysis to obtain a STR profile was not included. Manufacturers recommend a DNA amount of 1-2 ng to be used in their STR kits. The average quantity of recoverable DNA obtained from the swabs investigated ranged from 4 ng to 431 ng.

The retention of DNA by one type of swab material over the other is not significantly different. This confirms that DNA yields after 2 weeks storage represent the loss of DNA due to microbial activity and are not due to any difference in a particular swab material.

In addition, no viable microbes were identified in the forensiX evidence collection tube, whereas both viable fungi and bacteria were found on the Sarstedt Forensic swab. This supports the assumption that moisture-dependent microbial growth is the cause of the DNA degradation. Further model experiments, using the fast growing *P. putida*, showed that the decrease of intracellular ATP correlated with the drying performance of the forensiX tube indicating that fast drying to a level of 5% remaining water killed the bacteria. 

These findings are of particular importance for forensic samples containing a small quantity of DNA, for example, touch DNA samples: if it takes several hours or even days to dry such a sample to a level of 5% remaining water, then the quantity of recoverable DNA may be reduced to such an extent that no STR profile can be obtained. This study has shown the effect of degradation on substantial quantities of DNA when a sample is not rapidly dried and further studies are recommended to determine the effect of the drying rate on the quantity of recoverable DNA obtained from samples containing trace quantities of DNA.

The advantage of using the forensiX evidence collection tube with the active fast drying system (SafeDry desiccant) is that it allows the drying process to occur independent of the external conditions, that is, temperature and relative humidity. This is an attractive feature especially in warm and humid environments which enables easy handling of the sample once sealed in the collection tube.

The drying rate for a passive drying system is significantly slow in conditions of higher external relative humidity, meaning that a greater amount of water will remain on the swab for a longer period, thus increasing the likelihood of DNA degradation.

Fast drying DNA sample tools may improve the efficiency and cost effectiveness of forensic investigations not only by increasing the number of useable samples arriving at the forensic laboratory but also by ensuring that the majority of the samples received will indeed yield DNA profiles that can be used in the judicial process.

## 5. Conclusion

DNA sampling tools with rapid drying properties have shown to significantly improve the preservation of DNA. Such tools are expected to increase the quantity of DNA recovered from biological samples collected on a swab.

## Figures and Tables

**Table 1:** Summary of saliva-loaded tubes and storage conditions.

Sample no.	Type of storage tube	Storage condition
1–3	Microfuge tube (positive control)	Frozen (−20°C)

4–6	forensiX evidence collection tube; cotton swab with SafeDry desiccant	Frozen (−20°C)

7–9	Sarstedt forensic tube with ventilation membrane	Frozen (−20°C)

10–12	forensiX evidence collection tube without SafeDry desiccant	25 ± 2°C at 60% relative humidity

13–15	forensiX evidence collection tube; cotton swab with SafeDry desiccant	25 ± 2°C at 60% relative humidity

16–18	Sarstedt forensic tube with membrane sealed using Scotch tape	25 ± 2°C at 60% relative humidity

19–21	Sarstedt forensic tube with ventilation membrane	25 ± 2°C at 60% relative humidity

**Table 2:** Collection tubes and storage conditions for microbial growth assays.

Sample no.	Type of storage tube	Storage conditions
1–3	forensiX evidence collection tube	25 ± 2°C at 60% relative humidity; 14 days

4–6	Sarstedt forensic tube	25 ± 2°C at 60% relative humidity; 14 days

**Table 3:** DNA yields for the different storage methods.

Samples	Description of storage of saliva sample	Average DNA yield per swab (ng)	Standard deviation of results (ng)	DNA recovery relative to saliva controls
1–3	Microfuge tube, frozen, and control	455	47.4	100.0%

4–6	forensiX Swabs, frozen	328	75.4	72.0%

7–9	Sarstedt Swabs, frozen	246	20.3	54.0%

10–12	forensiX Swabs, without desiccant	22	4.5	4.8%

13–15	forensiX Swabs with desiccant	431	13.8	95.0%

16–18	Sarstedt Swabs, sealed	4	2.0	0.9%

19–21	Sarstedt Swabs, unsealed	56	3.3	12.3%

**Table 4:** Yield of saliva DNA from different swab materials (*n* = 3 × 2 = 6).

Samples	Description of storage of saliva sample	Conditions	Average DNA yield per swab (ng)	Standard deviation of results (ng)
1–3	Sarstedt swab (viscose)	Air dried: 56°C, 2 hr	331	84

4–6	forensiX swab (cotton)	Air dried: 56°C, 2 hr	309	76

**Table 5:** Colony numbers on SDA plates.

Sample type	forensiX evidence collection tube stored at 25°C, 60% relative humidity over 14 days			Sarstedt swab stored at 25°C, 60% relative humidity over 14 days		
Swab no.	**1**	**2**	**3**	**4**	**5**	**6**
No dilution	<1	<1	<1	5	>250	>250
1 : 10 dilution	<1	<1	<1	<1	39	19
1 : 100 dilution	<1	<1	<1	<1	5	<1

**Table 6:** Genus level identification of bacterial colonies obtained from Sarstedt tubes.

Genus	Number of colonies	Percent of total
*Klebsiella *	18	56%
*Citrobacter *	7	22%
*Raoultella *	6	19%
*Enterobacter *	1	3%

## Abbreviations

ATP: Adenosine-5′-triphosphate
cGMP: Current good manufacturing practice
cm: Centimetre
DNA: Deoxyribonucleic acid
mm: Millimetre
ng: Nanograms
pg: Picograms
*P. putida:*: * Pseudomonas putida*
%RH: Percentage relative humidity
STR: Short tandem repeat
SDA: Sabouraud dextrose agar
UV: Ultraviolet
ZHAW: Zurich University of Applied Sciences
*μ*L: Microlitre.
